# A Community Resource Navigator Model: Utilizing Student Volunteers to Integrate Health and Social Care in a Community Health Center Setting

**DOI:** 10.5334/ijic.5501

**Published:** 2021-02-05

**Authors:** Sahil Sandhu, Jacqueline Xu, Lillian Blanchard, Howard Eisenson, Carolyn Crowder, Veronica Sotelo Munoz, Connor Drake, Janet Prvu Bettger

**Affiliations:** 1 Trinity College of Arts & Sciences, Duke University, Durham, NC, USA; 2 Lincoln Community Health Center, NC, USA; 3 Sanford School of Public Policy, Duke University, Durham, NC, USA; 4 Duke Center for Personalized Healthcare, School of Medicine, Duke University, NC, USA

**Keywords:** social determinants of health, students, primary care, volunteer models, integrated health and social care

## Abstract

**Introduction:**

While unmet social needs are major drivers of health outcomes, most health systems are not fully integrated with the social care sector to address them. In this case study, we describe the development and implementation of a model utilizing student volunteer community resource navigators to help patients connect with community-based organizations (CBOs). We then detail initial implementation outcomes and practical considerations for future work.

**Methods:**

We used the Ten Essential Public Health Services Framework to guide program planning of a student “Help Desk” model for a community health center. Planning included a literature review, observation of exemplar programs, development of a CBO directory, and evaluation of the center’s patient population, clinical workflows, and data infrastructure. We piloted the model for two months. After pilot completion, we reviewed patient data to understand the feasibility of the student “Help Desk” model. We utilized planning and pilot execution materials, as well as pilot data, to develop and discuss practical considerations.

**Results:**

Design and implementation complemented ongoing social needs screening and referral to CBOs by center case managers. Patients were asked if they would accept telephone follow-up by volunteers two and four weeks after the clinic visit. Of 61 patients screened, 29 patients were referred for follow-up. Ninety percent were reached at least once during the follow-up period, and 48% of patients referred reported connecting to at least one CBO. Only 27% of patients required escalation back to case managers, and no emergency escalation was needed for any patients. Students, faculty advisors, and community health center frontline staff and leadership supported the scale up and continuation of the “Help Desk” model at the community health center.

**Discussion:**

Successful implementation required multi-sectoral collaboration, well-defined scope of practice, and data interoperability. Student volunteers are untapped resources to support integrated health and social care.

## Introduction

Addressing the social determinants of health (SDOH) is a critical step in reducing health disparities. The World Health Organization defines the social determinants of health as “the conditions in which people are born, grow, live, work and age” [1]. Specifically, examples of SDOH include housing status; food security; economic stability; education; neighborhood and physical environment; and community and social context [2]. SDOH and related health behaviors account for 80 to 90 percent of modifiable contributors to population health outcomes, while healthcare alone only accounts for 10 to 20 percent [3]. While health care alone is a relatively modest contributor to health, the United States spends nearly 20% of its gross domestic product on medical care compared with only 16% on social care, and achieves poorer health outcomes than peer countries [4]. This allocation of resources persists despite recent evidence that addressing individual social needs can improve health outcomes and expenditures [5,6,7,8,9].

In addition to limitations on financing social care, the systems of health and social care remain siloed. Research of care models integrating medical and social care demonstrate improvements in patients’ access to social services, patient satisfaction, health behavior, and healthcare utilization [7,10,11,12,13,14]. Achieving these outcomes requires a shift from the traditional biomedical model of health to establish closer collaborations with non-medical sectors in support of overall health and well-being. To implement person-centered integrated care models that aim to coordinate services across both health and social services, healthcare providers can start with a screening process for unmet social needs that enables targeted referrals to community or public government programs [15]. Research has demonstrated the feasibility of screening for unmet social needs as part of routine primary care visits [16,17,18,19]. There has been progress towards identifying strategies and workflows to integrate social needs screening and referral programs [20,21,22,23,24] based on a vision for organizing care that recognizes the importance of ‘moving upstream’ to promote health equity [25,26]. However, there is a lack of evidence describing the effectiveness of referring patients to community resources and the extent to which patients are able to access identified community resources [7,10].

Health systems often do not have the capacity to both screen for and respond to unmet social needs through follow up and ongoing case management [27]. One potential emerging solution to build capacity for integrated care is utilizing volunteers [28,29]. Volunteers are a low-cost and underutilized resource that health systems can leverage to help address patients’ unmet social needs while educating tomorrow’s workforce [10]. In a student volunteer based model, students can interact with patients to screen patients’ needs, refer them to community-based organizations, or provide short-term follow-up to motivate follow-through and problem solve barriers [30,31,32,33,34]. Evidence has shown that volunteers can help address patients’ routine social needs and allow clinic social workers and case managers to work at the top of their license, such as providing substance abuse and mental health treatment for higher-risk patients [30,32,33,34].

Volunteer models are typically adopted by larger health systems with resources to manage volunteer recruitment, training and oversight. However, safety-net community health centers, that serve uninsured and underserved individuals regardless of ability to pay, often lack resourced volunteer service programs yet are at the forefront of serving patients with unmet social needs and face greater resource constraints [35]. In our program pilot, we explored how student volunteers could support efforts to social needs screening, referral, and follow-up to improve integrated health and social care in a federally qualified health center. This integrated care case study is on our “Student Help Desk” model implemented at the Lincoln Community Health Center in Durham, North Carolina in partnership with Duke University. The aim of this case study is to provide a description of the process and practical considerations to implement a student volunteer “community resource navigator” model to address patients’ social needs.

## Description of the Care Practice

### Pilot Study Setting

Durham is a diverse mid-sized American city and county center. A nearly 20% increase in population over the past decade has contributed to financial development and investment [36]. However, economic growth has also been connected to growing income inequality and social stratification, contributing to increased disease prevalence (e.g., 14% diabetes and 65% obesity prevalence) [37] and social challenges (e.g. highest eviction rates in the state) [38].

The city is also home to Duke, a private university with a nationally prominent academic medical center. The initiators of this pilot study were four students at this academic institution, including two pre-medical undergraduate students in their 3^rd^ and 4^th^ year respectively, one 4^th^-year undergraduate psychology student, and one 2^nd^-year graduate student in public policy. The academic institution has a university-wide initiative to support interdisciplinary research teams of undergraduate students, graduate students, faculty and community partners tackling complex societal challenges [39]. As part of this credit-granting educational experience integrating research and civic engagement, students who led this pilot study were enrolled in a year-long course on “sustaining and scaling health innovation,” focusing on program planning, implementation, and evaluation.

The university project team formed an academic-community partnership with Lincoln Community Health Center, Inc. a non-profit organization operating since 1971 in Durham. As a federally qualified health center, Lincoln receives designated funding from the United States Department of Health and Human Services to serve as a safety-net primary care center for medically vulnerable and underserved populations. Lincoln operates at nine locations in Durham County and in 2019 saw 36,361 unique patients (over 127,341 patient visits). In 2019, 71% of Lincoln patients had incomes at or below 100% of the federal poverty level, approximately 57% of adults were uninsured, 89% were racial or ethnic minorities, and 51% said they were best served in a language other than English (mostly Spanish).

### Foundation for the Duke University-Lincoln Community Health Center Help Desk Model

Student volunteer models for social needs screening, referral, and follow-up have been developed and implemented across the country, but evaluation is sparse and translation to federally qualified health centers—where patient need is greatest—is lacking. To inform the development of our model, we examined the literature for best practices on volunteer-based models, shadowed existing program sites, and conducted key informant telephone interviews with leaders of current programs. We closely studied the original “Help Desk” program that trained college volunteers to screen patients for unmet social needs, refer patients to community resources, and follow-up via phone to ensure that connections were made [28]. This “Help Desk” model has scaled to locations across the country [31,40]. These existing models were predominantly implemented in pediatrics, emergency departments, or academic clinic settings. Interviewing both program leaders and student volunteers revealed facilitators and barriers to successful and sustainable program implementation, such as detailed program materials (e.g. telephonic scripts and documentation templates), clear volunteer scope of practice, and robust volunteer training. Site visits helped us to understand specific intervention components such as the populations served, governance structure, role of the volunteer, data infrastructure, and volunteer coordination and management. In the following section, we describe our team’s process for adapting and operationalizing program elements from other models to improve fit for our local context (***Table 1***).

**Table 1:** Examples of Adapted Model Components.


EXAMPLE ELEMENT	ORIGINAL MODELS	ADAPTATION

Personnel	Volunteers screen, refer, and follow-up [31,32,33,35,40]	Case managers screen and refer; volunteers follow-up at 2 and 4 weeks after visit

Setting	Academic emergency department [31]; Pediatric clinics [32], Academic primary care [33], County hospital [24],	Community health center adult primary care

Community Resource Referrals	Static, referral algorithm pathways [31,34]	Electronic, live spreadsheet directory updated through patient feedback

Screening tool	Local screening tool [31,32,33,35,40]	PRAPARE tool

Follow-up Script	Assess referral success and provide navigation [31]	Added questions on resource quality; Built-in motivational interviewing prompts


### Local Adaptation

Before committing to a full launch of our Help Desk program, we first focused on a shorter pilot to assess the feasibility of integrating student volunteers into our community health center’s existing process for addressing patients’ unmet social needs. Our measures of feasibility included the percentage of patients who agreed to follow-up calls from a student volunteer after their visit with a case manager, the percentage of patients reached on the phone by volunteers, and percentage of patient follow-up that volunteers could independently manage without further escalation to case managers. We adapted the “Ten Essential Public Health Services” framework (***Table 2***) to systematically plan and implement the Help Desk at Lincoln [41]. Commonly used for community program implementation, this framework outlines ten complementary goals to support a comprehensive infrastructure for public health priorities [42].

**Table 2:** Application of the 10 Essential Public Health Services Framework to Plan Help Desk for Implementation with a Community Health Center (CHC).


ESSENTIAL SERVICE	ORIGINAL FRAMEWORK COMPONENT	ADAPTED FRAMEWORK COMPONENT	EXAMPLE ACTIVITIES TO LOCALIZE HELP DESK

#1	Monitor health status to identify community health problems	Monitor social needs and health status of CHC patients	Create CHC patient profile sheet

#2	Diagnose and investigate health problems and health hazards in the community	Diagnose and investigate (1) CHC patient social barriers and (2) areas for improvement in CHC service delivery	Map existing case manager screening and referral workflow and practices

#3	Inform, educate, and empower people about health issues	Inform, educate, and empower people about unmet social needs	Present on social needs and Help Desk to university community at student showcase

#4	Develop policies and plans that support individual and community health efforts	Develop policies, infrastructure, and plans needed to implement Help Desk at CHC	Create volunteer workflows and build data infrastructure to collect information on Help Desk calls

#5	Enforce laws and regulations that protect health and ensure safety	Enforce laws and regulations that protect health and ensure safety	Onboard student volunteers at CHC to ensure compliance with safety and confidentiality regulations

#6	Mobilize community partnerships to identify and solve health problems	Mobilize community partnerships to identify and solve problems relating to unmet social needs	Attend county level meetings relating to social determinants

#7	Link people to needed personal health services and assure the provision of health care when otherwise unavailable	Implement Help Desk program and link patients to community resources that address social needs	Volunteers pilot Help Desk “follow-up” calls under supervision of case managers

#8	Assure a competent public health and personal health care workforce	Assure a competent volunteer workforce	Host weekly operational meetings and case reviews with volunteer base; identify and train next cohort of student leaders

#9	Evaluate effectiveness, accessibility, and quality of personal and population-based health services	Evaluate effectiveness, accessibility, and quality of Help Desk	Assess Key Performance Indicators noted in the logic model from pilot

#10	Research for new insights and innovative solutions to health problems	Research for new insights	


Over a three-month engagement period, the students, faculty director, medical director and members of the behavioral health team met to describe the target patient population [43], and map the existing workflow of social needs screening and referral. Prior to this pilot study, four case managers on the Lincoln behavioral health team screened patients for unmet social needs and referred them to resources offered internally by Lincoln or externally in the community, using the Protocol for Responding to and Assessing Patients’ Assets, Risks, and Experiences (PRAPARE) [44]. PRAPARE was developed by the National Association of Community Health Centers and partnering organizations, and includes a social needs screening tool and response protocol to assess patient non-medical needs, promote social service integration through community partnerships, and inform population health improvement.

Integrated into Lincoln’s electronic medical record, the screening tool was designed to assess a host of social factors related to health, including patients’ incarceration history, refugee status, language preference, race, ethnicity, level of education, veteran status, employment including migrant and/or seasonal farm work, income, health insurance, material security, housing status, location and stability, transportation options, social integration and support, stress, safety and domestic violence.

**Figure 1:**
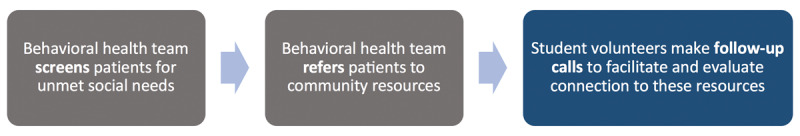
Community Health Center Help Desk Model.

Our thorough evaluation of existing practice revealed a significant opportunity for improvement: to support patients after their clinic appointment by encouraging follow-through with the CBO referral. The community health center did not have the capacity to consistently follow-up with patients and provide additional navigation support after initial screening and referral. This provided an opportunity for involvement of student volunteers. Our local model was developed with the intent to minimally disrupt existing practice while providing additional support to patients who were screened and referred (***Figure 1***).

The model we designed and piloted had three components. First, the behavioral health case managers screened patients either as part of a behavioral health appointment, after a primary care provider requested referral to behavioral health based on patient complexity, or when a patient was waiting for a primary care provider. Second, the case managers referred patients with identified needs to community resources. Third, our academic-community health center partnership enabled the creation of a volunteer “community resource navigator” role. In this role, student volunteers conducted follow-up calls with patients, discussed patients’ successes and challenges with accessing community services, co-developed plans to help patients access services for self-identified priority needs, and collected both quantitative and qualitative oral survey data on patients’ ability to use the community resources.

To support the behavioral health team with community resource referrals and to standardize knowledge of local services, students created a local community-based organization directory organized by PRAPARE social determinant of health domains (e.g. housing resources, food resources, transportation, legal aid, etc.). The directory included service descriptions, phone numbers, addresses, eligibility criteria, and languages served. The directory was designed to be used in real-time and updated continuously to incorporate feedback from patients, such as capacity of the organizations to serve more people.

To support development of the student volunteer community resource navigator role, our team designed and implemented a comprehensive volunteer training protocol. Training included didactic lessons on social determinants of health, sessions on motivational interviewing, and a patient engagement curriculum including role play scenarios; orientation to the data collection platform; training on data quality adherence; volunteer onboarding at the community health center; shadowing and peer observation; and evaluation by academic faculty. In order to standardize volunteer practices, we created telephone follow-up scripts for volunteers incorporating principles of motivational interviewing [45]. All volunteers were assessed on their ability to successfully implement the phone call protocol using a competency checklist adapted from the Association of American Medical Colleges 15-Core Competencies [46]. Further refinement and evaluation of the recruitment strategy and training curriculum is ongoing.

The students and behavioral health team co-designed Help Desk workflows and program materials. Case managers completed PRAPARE screening and referrals on paper, and student volunteers collected these forms for weekly data input using the secure REDCap electronic data capture tool hosted at Duke University [47,48]. Each student volunteer was paired with one case manager to maximize care coordination. Three volunteer-case manager pairs served primarily English-speaking patients while one-pair served Spanish-speaking patients. Volunteers then planned for follow-up calls with patients that received a community referral from their assigned case manager. Calls were scheduled for two and four weeks after their clinic visit during which the initial referral was made. During the call, volunteers asked patients if they were able to connect to the resource that was most important to them. If the patient was able to access the prescribed social service or community resource, the volunteer asked the patient to self-report perceived ease of use and usefulness of the service, to inform continuous curation of the CBO directory. If the patients were not able to connect with the service, volunteers assessed reasons for not connecting and subsequently: 1) reminded patients about the referral and motivated follow-through; 2) provided specific information for accessing the community service (e.g., eligibility, hours of operation, application process); and 3) connected the patient back to their case manager when needed, as defined by our escalation protocol based on urgency.

During the six-week follow-up period of the pilot, volunteers participated in two shifts each week between one and two hours. Students called patients remotely from a private “Help Desk” office in an academic building. During their first shift in the pilot, students were supervised by their faculty advisor. In subsequent shifts, students conducted follow-up calls on their own. Students had weekly check-ins with their assigned case manager to clarify referrals and discuss the care plan for the patient. The student volunteers had weekly operational meetings and weekly case review meetings to review each patient served and to ensure data quality. Patient feedback data was used to curate the existing directory of community-based resources and inform practice changes (e.g. refining the script) through discussions at case reviews.

**Figure 2:**
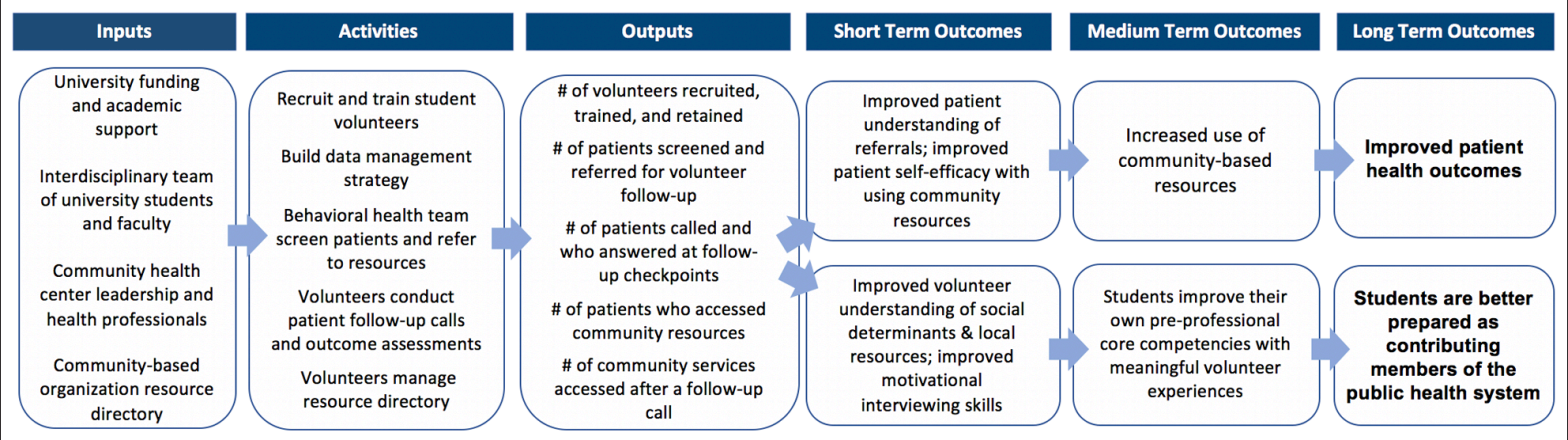
Logic Model.

A logic model further directed implementation and evaluation of our volunteer program (***Figure 2***) [42]. In this model, we highlighted the academic-community health center collaboration, the core activities, and the intended outcomes for both patients and student volunteer engagement. Our outputs were monitored weekly to support uptake. Specifically, we measured the number of patients who were screened, the number of patients who were referred to at least one resource, the number of patients who gave verbal permission for follow-up, and the number of patients reached within three call attempts. The pilot’s primary outcome measure was the number of patients who connected (or were in the process of connecting) to a resource prescribed based on an identified social need. Aggregate outcomes were tracked by the student volunteers and shared through standing monthly meetings with the community health center. During these monthly meetings, case managers also shared feedback on the pilot progress and highlighted opportunities for improvement.

Student program initiators received academic credit for participating in the Help Desk program for two semesters. The faculty advisor evaluated the student team through graded assignments scaffolded throughout the year: team charter; innovation analysis of similar Help Desk programs; action plan; program toolkit of protocols, forms, and templates; communication plan; and final report. Each semester, students also presented their progress publicly at university student research showcases. Students provided feedback on their experience to their faculty advisor and program funder through end-of-year surveys.

## Pilot Results

**Figure 3:**
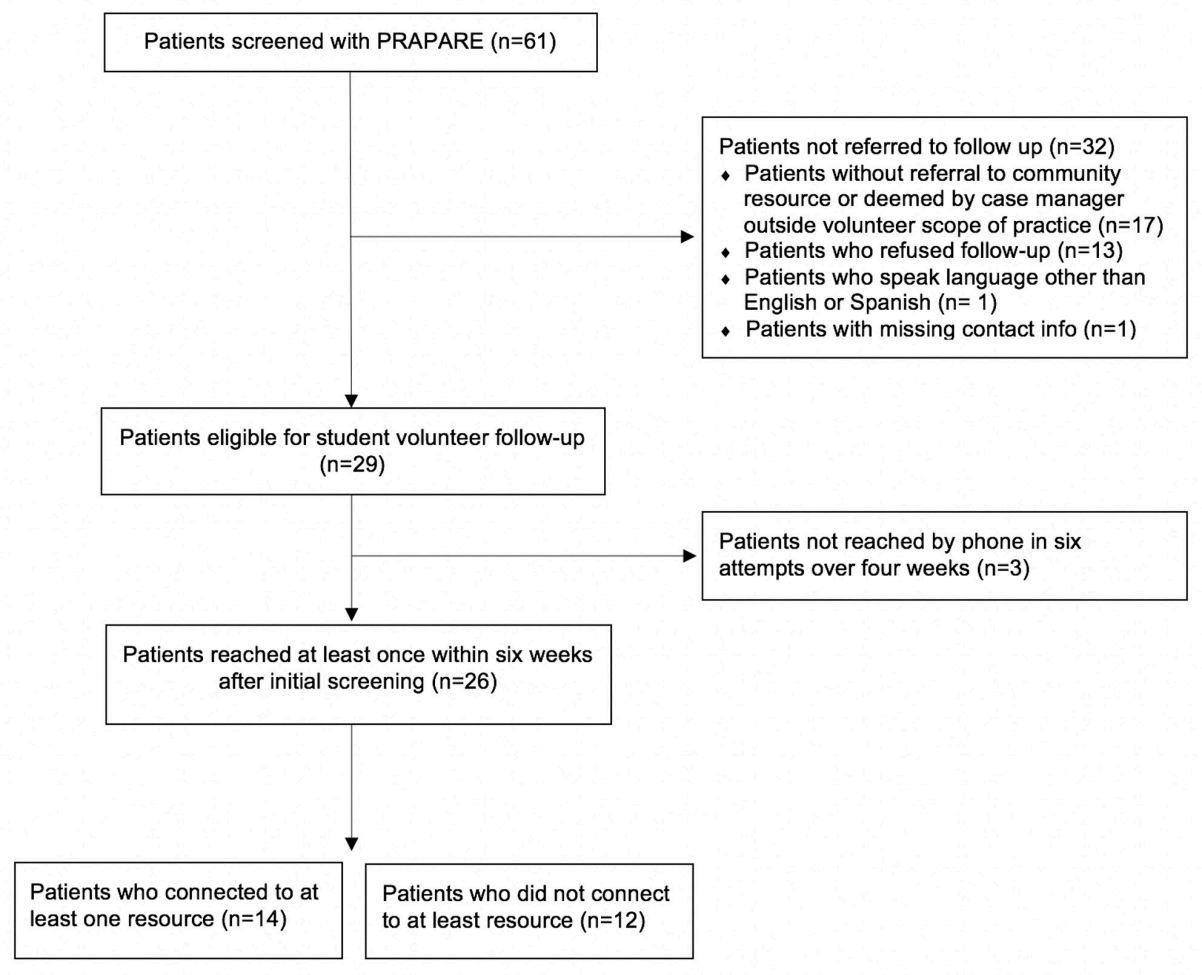
Flow Diagram of Pilot Study.

We conducted a two-month pilot of our community health center Help Desk model. This included a two-week screening and referral period, followed by six weeks of follow-up calls in the pilot period. Of the 61 patients screened for unmet social needs by the behavioral health case managers, 29 patients were referred to four Help Desk student volunteers for follow-up (***Figure 3***). Patients not referred (N = 32) constituted those without at least one community resource referral, those who refused to be contacted after the clinic visit, those with needs that student volunteers were not trained to manage (i.e. active domestic violence, severe mental health challenges), those who did not speak English or Spanish, or had missing contact information. Help Desk volunteers reached 26 patients (89%) by phone at least once within six weeks after their initial screening. Most (65%) of the patients reached were called on the first call attempt.

Of patients referred to services, the three most frequent unmet social needs were related to medical care (e.g. access to affordable medications or specialty care), food insecurity, and financial assistance. Eighty-three percent of patients had referrals to community and social services for at least one of these top three referral areas. Fourteen patients (48%) reported accessing at least one of the recommended community resources. Barriers for patients unable to connect to the resources recommended included lack of time, lost contact information, need to clarify service application process, compromised physical health, major life events that took precedence, and failed contact with community-based organizations.

Out of the 26 patients reached by volunteers, only seven patient cases were escalated for additional input or action from the behavioral health case manager. In the majority of these seven cases, the case manager was included primarily to encourage or reinforce the importance of the community resource referral. None of the patients presented with an emergency during follow-up that student community resource navigators needed to immediately escalate.

## Discussion

Social needs and related health behaviors are major drivers of health outcomes [3]. Federally qualified health centers care for some of the most vulnerable people in the United States yet often lack the capacity to fully support and coordinate their patients’ follow-through care. In this descriptive case study, we outline our structured process for planning and implementing a student volunteer program to help patients navigate community resources for their social needs. Our efforts underscore key considerations that may help other organizations develop academic-community partnerships to support integrated health and social care.

We found the following principles to be integral to successful implementation and program feasibility: 1) multi-sectoral collaboration; 2) well-defined scope of practice; 3) data systems to produce timely and actionable reports; and, 4) mechanisms for sustainability including training and onboarding, supervision, case conferences, and quality improvement.

Multi-sectoral collaboration was integral to building this student volunteer Help Desk for the community health center. Research and design was led by a team of Duke University students and their faculty mentors through a university-sponsored program to encourage interdisciplinary research and program implementation. While other student models were completely designed by research and clinical staff [31,32,34], the planning process for this program, (e.g. creation on program materials, CBO directory creation, and database) was driven by students as part of a for-credit course. Following an academic course schedule with clear, graded deadlines helped keep the project planning on-track and created a culture of accountability. Unlike large academic medical centers, community health centers may not have the resources to develop and sustain a volunteer program on their own. Nonetheless, although this university-affiliated team led the intervention adaptation and refinement, they relied heavily on partnership and co-design with Lincoln Community Health Center leadership. Future evolutions of this model will need to both expand on Lincoln’s existing relationships with community-based organizations and consider state-level efforts to address social determinants of health [49]. For example, processes will need to be developed for integrating with the statewide community resource platform designed to improve coordination and communication between health and human services [50]. Similarly, future research should evaluate the cost and cost-effectiveness of this model to inform its scale as a population health strategy.

The volunteer’s scope of practice was context specific and developed through multiple iterations of stakeholder feedback. Translation to other clinics will require local evaluation of workflow in order to achieve the similar success. Our project team designed a community resource navigator role that freed up behavioral health case managers to pursue other responsibilities and maximize volunteer impact. Compared to other Help Desk models in which volunteers conduct SDOH screening and referrals [32,33,34,40], our program’s volunteers focused exclusively on follow-up. Such an approach aimed to lower the risk of the pilot by simplifying volunteer responsibilities, and thus increase program feasibility. In settings with other provider models or universal self-screening, continuity with student volunteers may require new workflows. There may be benefit in comparing the scope of practice for the student’s community resource navigator role with that of other navigators—peer navigators, community health workers, link workers—in order to establish a workforce model for wide-scale implementation [15,23].

One of the largest challenges in implementing this model was designing secure and actionable data systems. Risk compliance made it impossible for volunteers to work within the community health center’s electronic health record system resulting in double data entry of the data obtained using PRAPARE screenings. Generating reports to track progress and identify areas for improvement required access to the electronic health record to compare social needs information with health data not captured in PRAPARE (e.g., health status, comorbidities, etc.). In the future, this could be improved by enabling electronic data capture of the initial social needs screen that automatically populates both the health record and the student volunteer’s data privacy compliant database.

Building sustainability beyond the pilot primarily centered on the student workforce. Recruitment, onboarding and training had to commence during the pilot period in order for the volunteer program to be resourced appropriately for the summer months when students often leave the area. Strategies to address turnover beyond the academic year transitions included creating clear expectations for volunteer commitment and having at least one clinical champion and one volunteer coordinator to ensure continuous Help Desk operations through holidays and academic calendar breaks. The investment of university resources including faculty mentors, student project leads, space and resources for volunteers and their training were essential. A weekly seminar course to teach on topics of social determinants, health service delivery, evaluation, quality improvement, and health policy complemented the efforts and contributed to the students’ learning and engagement.

Our pilot data also support the feasibility of integrating student volunteers as community resource navigators, according our feasibility measures of rate of patient refusal for follow-up, rates of patient contact via phone, and rate of escalation of patients to case managers. Only 21% of patients screened refused to be contacted by student volunteers after the clinic visit, and 89% of referred patients responded to student call attempts and voicemails on follow-up. Such data suggests that patients are open to follow-up calls from volunteers and can be consistently reached via phone. Further, the majority of patient interactions could be resolved by the student volunteers directly. Student volunteers reconnected 27% of patients with their case managers for additional support, and case managers reported this referral stream as acceptable and not burdensome. Of the patients reached by student volunteers, no emergency escalation was needed. This indicates that patients’ needs fell within the scope of practice of the navigators and limited the need for additional clinical resources due to escalation.

After the pilot program, clinic leadership, case managers, the student team, and faculty advisors agreed the student Help Desk program was feasible and committed to sustain the program at the community health center. Case managers felt that the Help Desk program did not significantly burden their workload and allowed for follow-up that the clinic could not otherwise provide. Clinic leadership was motivated to collect more follow-up patient data to assess the effectiveness and cost-effectiveness of SDOH screening and referrals in order to better inform decisions related to long-term investments in the program and potential expansion to satellite clinics. Students and faculty advisors felt the experience would be a worthwhile experience for future students, and received funding from the university to continue to scale the program operations and conduct further evaluations.

Between the pilot in spring 2019 and fall 2020, 19 additional student volunteers have been recruited and trained as community resource navigators. These students included medical students, nursing students, and undergraduate students from social science, public policy, pre-dental, and pre-health backgrounds. Since the pilot program, over 1900 patients have been screened for social needs and over 500 patients have been reached for follow-up by Help Desk volunteers. While our pilot study focused on feasibility, our team’s future analyses will focus on workforce development, implementation, and effectiveness.

The Help Desk student volunteer model for community health centers is a valuable addition to the healthcare system and to society because it provides young adults interested in health careers with firsthand exposure to social determinants of health, collaborative evaluation, and problem solving. By design, our model included interdisciplinary and interprofessional engagement of students in different levels of learning (undergraduate and graduate) and fields of study related to healthcare. Our training protocol incorporated experiential, project-based learning and community engagement to expose students to the connection between unmet social needs and health. Our Help Desk model contributes to the growing body of research on educating students in social determinants, population health, and implementation science [51,52,53].

## Conclusion

Due to limited staff and resources, federally qualified health centers staff often feel challenged to respond to positive social needs screens, track outcomes of past referrals, and keep resource lists up to date [18]. Previous studies have explored the feasibility of screening for unmet social needs in the community health center setting [19,27,54], but have lacked discussion as to how community providers might build this capacity to conduct screening and follow-up.

In our case study, we describe in detail a structured process in which university students and their advisors were able to partner with a community health center to launch volunteer resource navigator programs. Specifically, we highlight key steps in our planning and implementation steps, such as studying similar models through literature review and observation, developing a resource directory, and evaluating the center’s patient population, clinical workflows, and data infrastructure. We also highlight four principles key to program feasibility. Our pilot data further reinforces the feasibility and sustainability of this model. We recommend that community health centers in areas with institutions of higher education consider these academic-community collaborations to increase capacity to serve patients’ needs and provide patient-centered care beyond individual clinic appointments. Trained student volunteers are an untapped resource that provide a low-cost and high-yield solution and can improve integrated care.
